# Surgical Management of Degenerative Lumber Spine Disease: A Comparative Study of Microdisectomy Versus Open Disectomy

**DOI:** 10.7759/cureus.90178

**Published:** 2025-08-15

**Authors:** Abdul Moez, Saddam Hussain, Murad Ali, Sohail Ahmed, Adnan Khan, Muhammad Rizwan Umer, Hifza Ishtiaq, Amna Akbar, Palwasha Noor, Muhammad Iftikhar Khattak, Ayesha L

**Affiliations:** 1 Neurosurgery, Ghurki Hospital, Punjab, PAK; 2 Neurosurgery, Combined Military Hospital, Rawalakot, PAK; 3 Trauma and Orthopaedics, Ayub Teaching Hospital, Abbottabad, PAK; 4 Gastrointestinal Surgery, Yangtze University, Jingzhou, CHN; 5 Gastrointestinal Surgery, Yangtze University, Yangtze, CHN; 6 Trauma and Surgery, Royal Sussex County Hospital, Brighton, GBR; 7 Public Health, Abbas Institute of Medical Sciences, Muzaffarabad, PAK; 8 Trauma and Orthopaedic Surgery, Sarosh Hospital Diagnostic Center, Muzaffarabad, PAK; 9 Public Health, Health Services Academy (HSA), Islamabad, PAK; 10 Research and Development, Celestial and Dimanche, Muzaffarabad, PAK; 11 Medicine and Surgery, Dow University of Health Sciences, Karachi, PAK

**Keywords:** complications, degenerative lumbar spine disease, functional recovery, microdiscectomy, open discectomy, oswestry disability index, pain relief, patient satisfaction

## Abstract

Background

Degenerative lumbar spine disease (DLSD) is a prevalent condition that significantly impacts quality of life. Surgical interventions, including microdiscectomy and open discectomy, are commonly used to treat symptomatic lumbar disc herniation. However, the comparative effectiveness of these procedures remains a subject of ongoing research.

Objective

This study aims to compare the clinical outcomes of microdiscectomy and open discectomy in patients with degenerative lumbar spine disease, focusing on pain relief, functional recovery, complications, and patient satisfaction.

Methods

A total of 600 patients (309 microdiscectomy, 291 open discectomy) were included in this prospective cohort study. Data were collected on demographic characteristics, preoperative and postoperative pain scores, functional recovery (measured by Oswestry Disability Index), and complications. Logistic regression and multiple linear regression models were employed to predict outcomes, and random forest analysis was used to identify key predictors of complications.

Results

Microdiscectomy was associated with significantly better postoperative outcomes, including reduced pain (213/309 (69%) vs. 146/291 (50%)), faster recovery (mean recovery time 5.2 weeks vs. 7.6 weeks), fewer complications (46/309 (14.9%) vs. 67/291 (23%)), and higher patient satisfaction (101/309 (32.8%) vs. 73/291 (25%)). Additionally, microdiscectomy also resulted in larger overall improvements in functional recovery and mobility scores compared to open discectomy.

Conclusion

Microdiscectomy provides superior results in pain relief, recovery time, and complication rates compared to open discectomy. This supports recommending microdiscectomy (with proper patient selection) for most patients with lumbar disc herniation.

## Introduction

Degenerative lumbar spine disease (DLSD) is one of the most common health conditions and a significant source of impairment in function and quality of life in the older adult. It can be viewed as a syndromic entity comprising of ratable degeneration of the intervertebral discs, facet joints, and anatomic components of the lumbar spine [1]. With progressive degeneration of disc and a complex interaction of factors, outcomes can be classified as chronic pain, radiculopathy, and compromised motion. Age is the major contributor, as most older adults experience dehydration and loss of elasticity of the intervertebral discs, culminating in diminished disc height and an increase in bulging and herniated discs [2]. Studies indicate that 90% of individuals about 60 years of age have imaging evidence of disc degeneration, but only a small number will have meaningful discomfort. Other possible risk factors for DLSD include mechanical stress, obesity, repetitive trauma, poor posture, and a sedentary lifestyle. When included with age itself, they can serve to accelerate the degenerative processes at the level of the lumbar spine and is associated with diminished quality of life for many older adults [3].

In the United States, symptomatic lumbar disc herniation (LDH) affects about 1%-2% of the adult population [1] and is a presentation of degenerative lumbar spine disease (DLSD). In cases of symptomatic LDH where conservative treatment options, such as physical therapy, medications, and lifestyle modifications, do not successfully relieve symptoms, surgical intervention may be necessary [4]. Microdiscectomy and open discectomy are two of the most commonly performed surgical procedures for symptomatic lumbar disc herniation. They share common ground in that they target the disruption of the herniated disc material and decompression of the spinal nerves. While both surgical procedures are effective in relieving symptoms and restoring function, there are substantial debates on which surgical approach offers the best clinical outcomes with regard to pain relief, functional recovery, and complications [5].

Open discectomy is the traditional approach that requires a longer incision and provides direct access to the affected disc. The open discectomy procedure is designed to allow the surgeon to remove the herniated disc material, but it usually involves a longer recovery time, more postoperative pain, and a greater risk of complications, including infection, nerve injury, and blood loss [6]. Studies show that recovery after open discectomy was on average four to six weeks with complications occurring in up to 15% of patients. On the other hand, microdiscectomy is a minimally invasive technique that uses a smaller incision and specialized instruments to excise the herniated disc material. Microdiscectomy offers several advantages, including less recovery time, less postoperative pain, and lower complication rates [7]. Evidence has shown that most patients after microdiscectomy return to normal activities in about two to four weeks, which is much shorter than the open discectomy approach. In addition, complications were significantly lower with microdiscectomy surgery, occurring in 5%-10% of patients. Nevertheless, microdiscectomy is not appropriate for all types of herniation, specifically larger or central disc herniations typically use the open technique, as this allows adequate removal of the disc material [8].

Though there is an increasing amount of evidence that supports the benefits of microdiscectomy, there is still a lack of evidence comparing microdiscectomy with open discectomy in a way that takes into account any patient-specific factors such as age, comorbidities, and characteristics of the disc herniation [9]. Many of the published studies evaluated each technique independently and many incorrectly compared techniques without consideration for patient-specific factors [5]. Additionally, most of the evidence published focuses on short-term outcomes, with little information comparing long-term recovery, reherniation, and patient satisfaction [4,10]. This study aims to address these gaps by comparing the clinical outcomes of microdiscectomy and open discectomy for patients diagnosed with degenerative lumbar spine disease. The aim of this study is to measure postoperative perceived pain levels, functional recovery, complication rates, and long-term outcomes for each surgical technique in order to understand which might be the most effective therapy for the treatment of DLSD, which has significant healthcare costs and burden to patients who suffer from it [11].

This study mainly aims to find out which surgical method, microdiscectomy or open discectomy, produces better clinical outcomes in managing patients with degenerative lumbar spine disease. The specific aims of the study are to assess the postoperative pain relief, time to return to normal function, and the occurrence of complications such as infection, neurological injury, and recurrence of disc herniation. The study will also review the demographic variables, comorbid conditions, and characteristics of the herniation that may be important in influencing the choice of surgical technique. This study is aimed at evidence-based recommendations that can help guide decision-making in our practice to optimize outcomes for individuals receiving surgical interventions related to DLSD.

## Materials and methods

Study design

This study applied a prospective, observational, comparative cohort analysis to evaluate the outcomes of two commonly performed procedures, microdiscectomy, and open discectomy, to treat DLSD. By observing and comparing both groups, the study permits a valid comparison of the postoperative outcomes with attention focused on the differences related to pain reduction, functional improvement, complication rates, and recovery times. The study was designed to mirror real-world clinical practices with a minimal bias from interventions, so valid information was provided about the comparative effectiveness of these two procedures in real surgical practice. Potential sources of bias included selection bias (surgeon preference and patient suitability influencing surgical approach) and information bias (variation in documentation of preoperative and postoperative outcomes). While regression models adjusted for measured confounders such as age, BMI, and baseline pain scores, residual confounding from unmeasured factors cannot be excluded. Future studies using randomized controlled designs or propensity score matching could further mitigate these biases.

Population

The sample comprised 600 adult subjects with lumbar disc herniation, with the diagnosis of degenerative disc disease. We included adults aged 20-85 years, with symptomatic DLSD who had not responded to conservative treatment for at least six weeks post-treatment initiation and who were aware of the subject matter of the study. We excluded individuals with prior lumbar surgery, spinal tumors or infections, severe systemic disease and patients with congenital spinal deformities. We divided the subjects into two cohorts relative to the surgical procedure performed: the microdiscectomy group (n=309) and the open discectomy group (n=291). All patients received treatment at a tertiary care centre, and follow-up, some continuing up to 12 months post-operatively, was conducted to assess outcomes.

Data collection

Data collection utilized a retrospective chart review combined with a prospective follow-up record. The collected demographics included age, sex, BMI, occupation, education, income, marital status, smoking history, and alcohol use history. The clinical characteristics collected were pre-operative symptoms, pain level, duration of symptoms, physical exam results, co-morbidities, and previous treatment. The diagnostic characteristics collected were radiological characteristics, reports regarding magnetic resonance imaging (MRI) findings, and neurological examinations. The perioperative variables collected included the type of discectomy, operative time, intraoperative complication, and length of hospitalization. The postoperative forms contained the following information: recovery time, number of complications, pain improvement, ambulation score, and functional disability scores, especially the Oswestry Disability Index (ODI), which was originally developed by Fairbank and Pynsent (2000). The index was accessed via National Institutes of Health (NIH)-hosted literature for non-commercial, academic research purposes. No modifications were made to the instrument, and it was used within the scope of fair use for observational analysis. Patients were assessed on postoperative months one, three, six, and 12 to document improvement in pain and disability scores, and any complications related to surgical or inpatient care. To ensure data quality, double-entry verification was applied during the data entry process, with discrepancies resolved through cross-checking of original medical records. Outliers and implausible values (e.g., negative recovery times, physiologically implausible BMI values) were reviewed by two independent investigators, and corrections were made where verifiable. Missing data were addressed using predefined imputation rules for variables with <10% missingness; otherwise, complete case analysis was applied.

Outcome measures

The study determined outcomes that were classified as primary and secondary. The outcomes that were identified as primary were postoperative pain relief and functional recovery, which were measured using the Visual Analog Scale (VAS) and ODI, respectively. VAS and ODI scores were recorded preoperatively and at every follow-up to assess a patient’s improvement. The secondary outcomes that were observed were complication rates, specifically surgical site infection, dural tear, recurrent herniation, and readmission. Patient satisfaction was recorded at prescribed intervals through a standardized postoperative satisfaction survey, while recovery time was considered as a period of time that a patient required to return to their normal daily activity or back to work.

Exploratory data analysis (EDA)

Before any formal modeling using statistics were carried out, exploratory data analysis was conducted to assess the distribution of the data, discover outliers and assess if the data were complete. Missing data was addressed by using imputation techniques for variables where there was less than 10% missingness, and complete case analysis for others. Categorical variables were described as frequency distributions and continuous variables were described as mean (±standard deviation). In exploratory data analysis, continuous variables such as pain scores, BMI and age were summarized using histograms and boxplots; categorical distributions such as gender and comorbidities were summarized using bar graphs. A correlation matrix was constructed to understand the linear relationships between continuous predictor variables and to explore multicollinearity before conducting regression analysis. T-tests and chi-square tests were used during exploratory data analysis to conduct a preliminary examination of any potential group differences.

Predictive modeling

To evaluate factors influencing surgical outcomes, predictive modeling techniques were applied. Logistic regression models were used to predict binary outcomes such as the occurrence of postoperative complications and patient satisfaction. These models included predictors such as surgical type, age, BMI, comorbidities, and preoperative pain levels. Additionally, multiple linear regression models were used for continuous outcomes, including recovery time and post-discectomy pain scores. Predictor variables were chosen based on their clinical relevance and statistical significance from EDA. Furthermore, a random forest model was applied to capture non-linear interactions and enhance the robustness of predictions. Variable importance plots generated from this model helped identify which clinical or demographic features were most influential in predicting patient recovery.

Tools and software

Three main tools were employed for data processing and analysis: SPSS, Excel, and Python. SPSS 27.0 (IBM Corp., Armonk, NY, USA) was used for initial data exploration, hypothesis testing, and statistical comparisons including t-tests, ANOVA, chi-square tests, and logistic regression. Its user-friendly interface made it suitable for analyzing categorical and normally distributed continuous data. Excel (Microsoft Corp., Redmond, WA, USA) served as a supportive tool for data entry, formatting, basic sorting, and preliminary chart creation, particularly useful during the data cleaning phase. Python (Python Software Foundation, Wilmington, DE, USA), with libraries such as pandas, NumPy, matplotlib, seaborn, and scikit-learn, was used for more advanced tasks, including data preprocessing, exploratory visualizations, and machine learning modeling.

Ethical considerations

While the retrospective collection of clinical and demographic data were taken from patient charts, the prospective components of the data such as the postoperative follow-ups and satisfaction surveys required direct interaction with patients. For the postoperative follow-ups, informed written consent was obtained at the time of surgical consultation or during hospital admission prior to inclusion into the postoperative monitoring data collection. Patients were informed of the entire project, including study details and those who agreed to participate allowed for monitoring and collection of data in conjunction with the completion of their postoperative follow-up, including 12 months following surgery. The study protocol was approved by the Institutional Review Board (IRB) of the affiliated hospital.

## Results

Demographic characteristics

**Figure 1:**
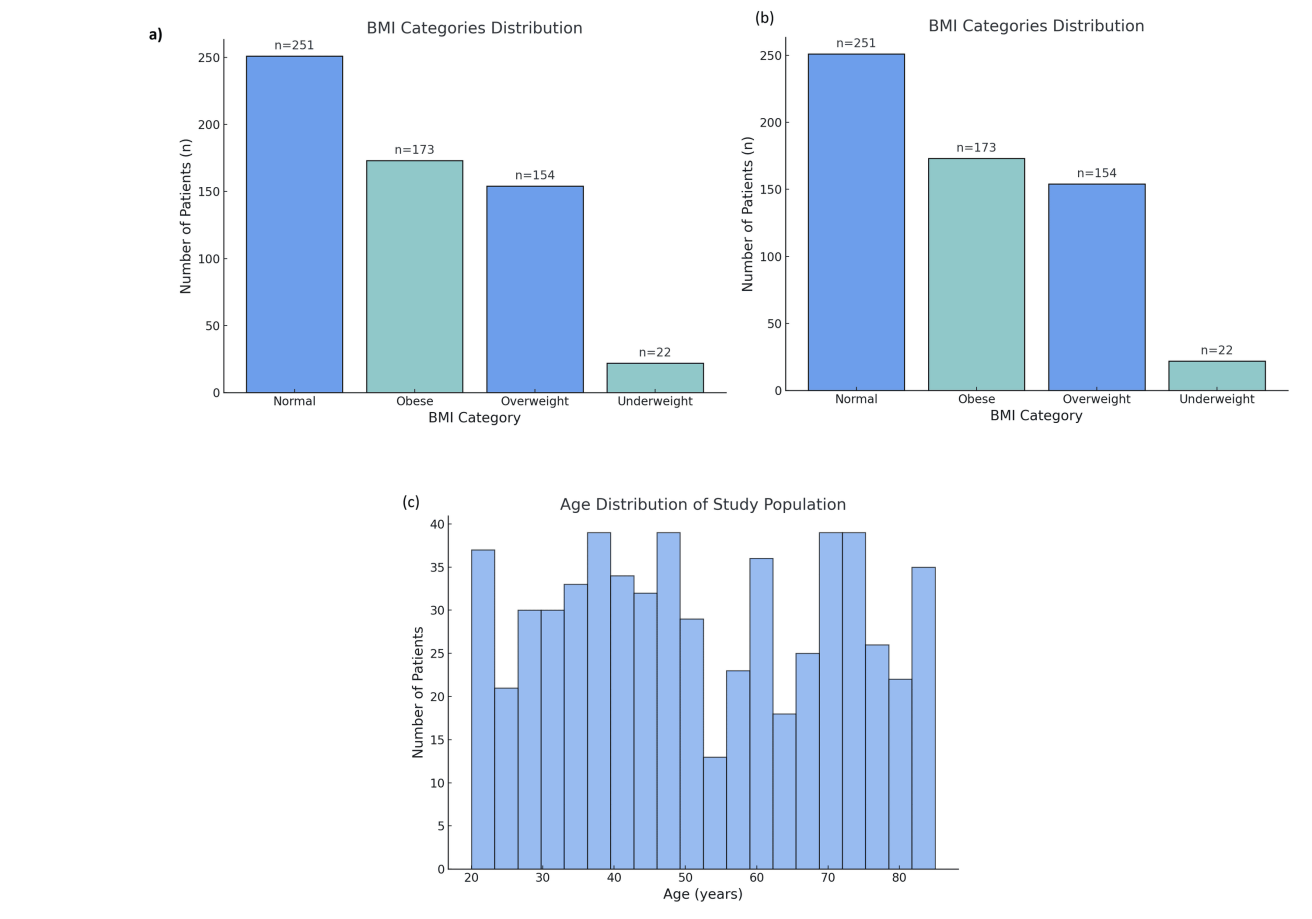
Distribution of BMI and Age Across the Study Population: (a) Distribution of BMI categories across the study population, with normal BMI (n=251), obese (n=173), overweight (n=154), and underweight (n=22) patients. (b) Similar BMI category distribution as in (a), illustrating the breakdown by BMI classification, with higher prevalence in the normal BMI category. (c) Age distribution of the study population, showing a relatively balanced spread across various age groups. The majority of patients are in the range of 40-50 years and 60-70 years, with a peak in patient numbers in the 70-80 year age group.

A total of 600 patients were included in the study: 309 (51.5%) underwent microdiscectomy and 291 (48.5%) underwent open discectomy. Among all participants, 318 (53.0%) were men and 282 (47.0%) were women. The mean age across the population was approximately 50 years, with microdiscectomy patients being slightly younger on average (mean=48 years) compared to those in the open discectomy group (mean=52 years). In terms of BMI, 185 (59.9%) microdiscectomy patients and 140 (48.1%) open discectomy patients were classified as overweight (BMI=25-30), suggesting a higher prevalence of excess weight in the former group. Regarding preoperative mobility, 173 (56.0%) in the microdiscectomy group reported moderate mobility impairment (score 4-6), while 180 (61.9%) in the open discectomy group had a similar level of mobility impairment. Ethnicity was homogeneous in both cohorts, with all participants identified as Pakistani, reflecting the regional demographic of the study (Figure 1).

Preoperative pain and mobility scores

**Figure 2:**
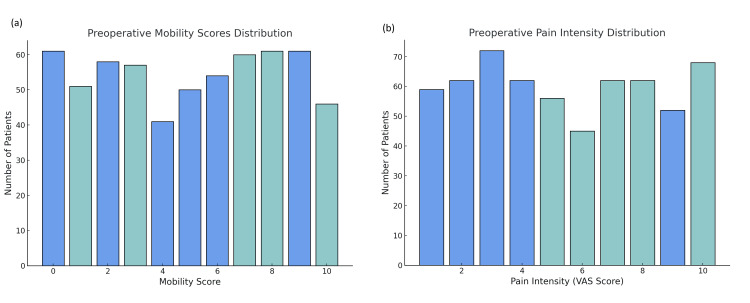
Preoperative Mobility and Pain Scores: (a) Distribution of preoperative mobility scores across the study population. The majority of patients reported mild to moderate mobility impairment, with a significant number falling within the mobility score range of 0-2 and 8-10. (b) Distribution of preoperative pain intensity as measured by the Visual Analog Scale (VAS). Most patients reported moderate pain intensity, with higher concentrations in the VAS score range of 2-4, though a notable number of patients also reported more severe pain (VAS 8-10).

Preoperative pain intensity, measured by VAS, was moderate (score 4-6) in 164 (53.1%) microdiscectomy patients and 175 (60.1%) open discectomy patients. The average pain score for the microdiscectomy group was 5.22, compared to 5.71 for the open discectomy group. Mobility scores prior to surgery showed that 127 (41.1%) microdiscectomy patients had scores between 7 and 9, indicating moderate impairment, while 110 (37.8%) open discectomy patients scored similarly, suggesting slightly poorer baseline function in the latter group (Figure 2).

Surgical characteristics

**Figure 3:**
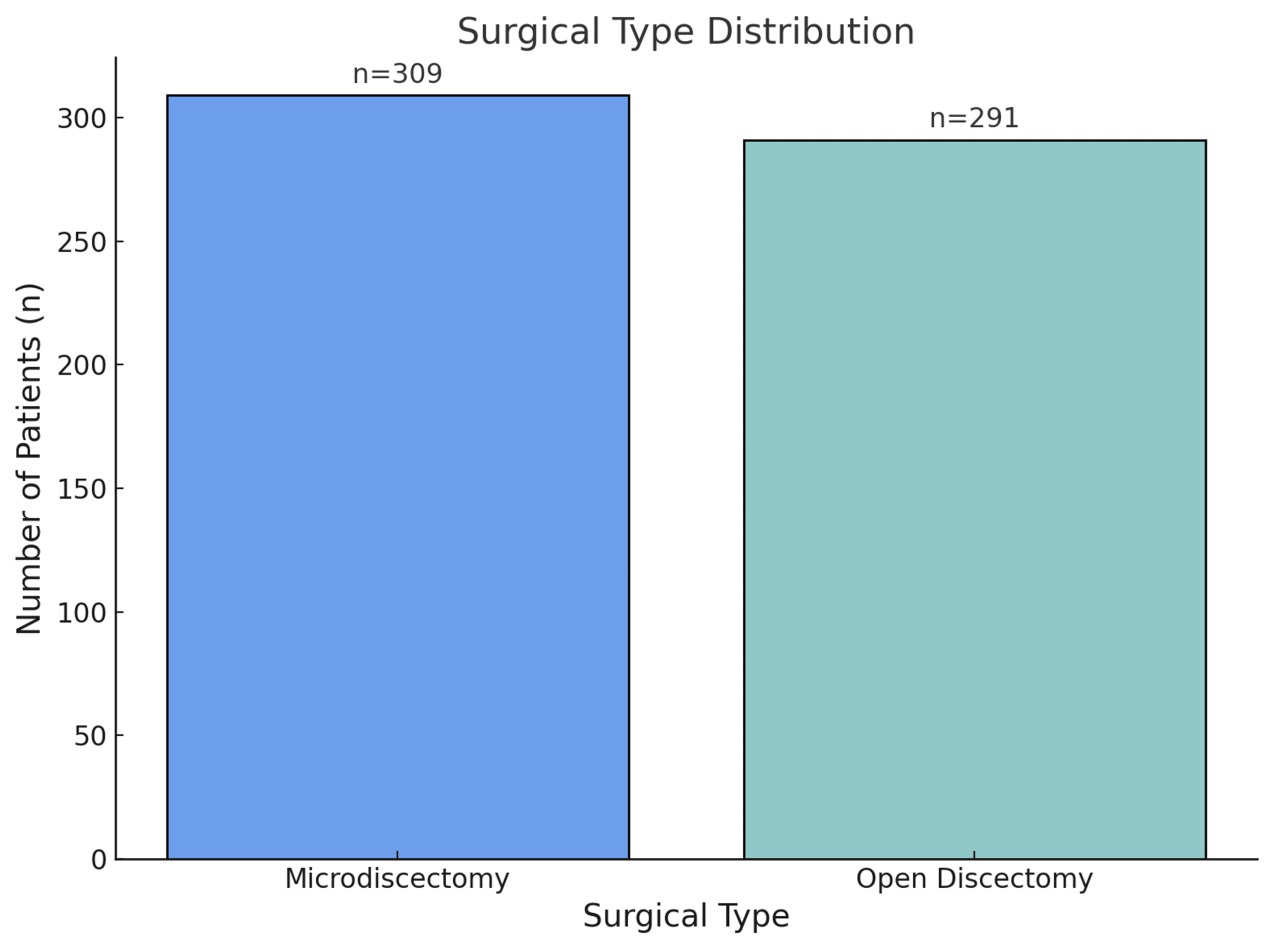
Surgical type distribution of the study population, showing the number of patients who underwent microdiscectomy (n=309) and open discectomy (n=291). Blue bars represent microdiscectomy and green bars represent open discectomy, indicating the distribution of patients between the two surgical approaches.

Out of the total procedures, 309 (51.5%) underwent microdiscectomy and 291 (48.5%) underwent open discectomy. Microdiscectomy was typically performed using minimally invasive approaches, with an average operative time of 70 minutes (SD=12) compared to 90 minutes (SD=16) in open discectomy. This difference was statistically significant (p<0.05) (Figure 3).

Postoperative pain and recovery

**Figure 4:**
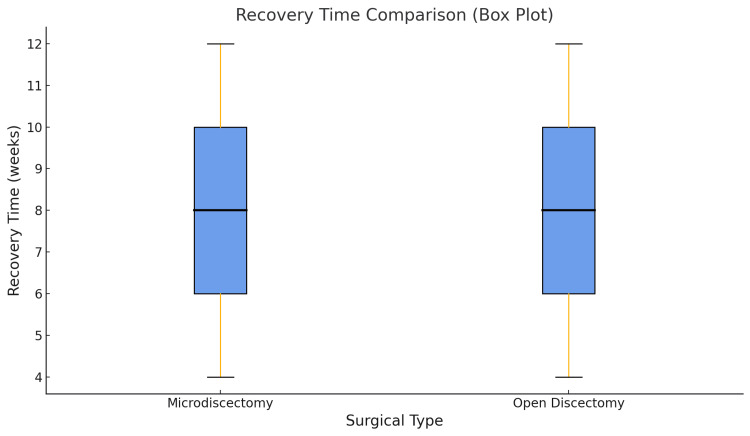
Recovery time comparison (box plot) illustrating the differences in recovery time between microdiscectomy and open discectomy patients. The plot shows that the recovery time for microdiscectomy patients is generally lower, with a median of around eight weeks, while open discectomy patients have a higher recovery time, with a median of approximately nine weeks. The spread (range) of recovery time is also wider for the open discectomy group, indicating more variability in the time taken to return to normal activities. The microdiscectomy group shows a more concentrated recovery period, primarily between six and nine weeks.

Following surgery, 213 (69.0%) microdiscectomy patients reported VAS pain scores of 0-3, indicating substantial relief, compared to 146 (50.2%) in the open discectomy group. The mean postoperative VAS score was 2.3 for microdiscectomy and 3.8 for open discectomy (p<0.01). Recovery time was notably shorter for microdiscectomy patients. A total of 173 (56.0%) microdiscectomy patients resumed daily activities within 4-7 weeks, whereas 140 (48.1%) open discectomy patients required 8-12 weeks. The mean recovery time for the two groups was 5.2 and 7.6 weeks, respectively, and the difference was significant (p<0.01) (Figure 4).

Disability and functional recovery

**Figure 5:**
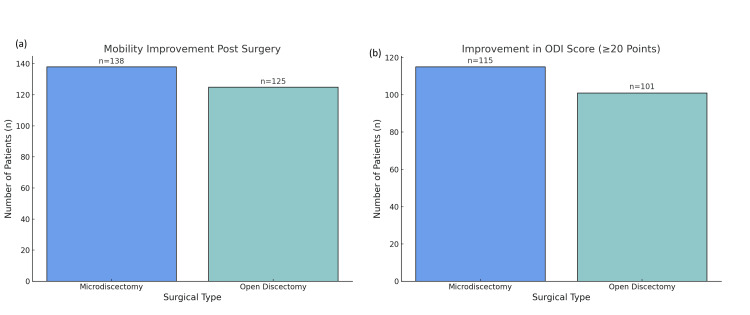
Postoperative improvements in functional mobility and ODI scores: (a) Mobility improvement post surgery: Distribution of patients showing mobility improvements after surgery, with 138 patients in the microdiscectomy group reporting significant improvement compared to 125 in the open discectomy group. (b) Improvement in ODI score (≥20 points): Number of patients showing significant improvement in Oswestry Disability Index (ODI) score (≥20 points): 115 patients from the microdiscectomy group showed improvement, while 101 patients from the open discectomy group demonstrated similar progress.

Improvements in function were assessed using ODI. A score reduction of ≥20 points was observed in 149 (48.2%) microdiscectomy patients versus 96 (33.0%) patients in open discectomy. The average ODI improvement was 20 points in the microdiscectomy group and 14 points in the open discectomy group (p<0.01). Mobility gains were also more prominent in the microdiscectomy cohort, where 216 (69.9%) showed major functional improvement by the third month postoperatively, compared to 169 (58.1%) in the open discectomy group (p=0.04) (Figure 5).

Postoperative complications

**Figure 6:**
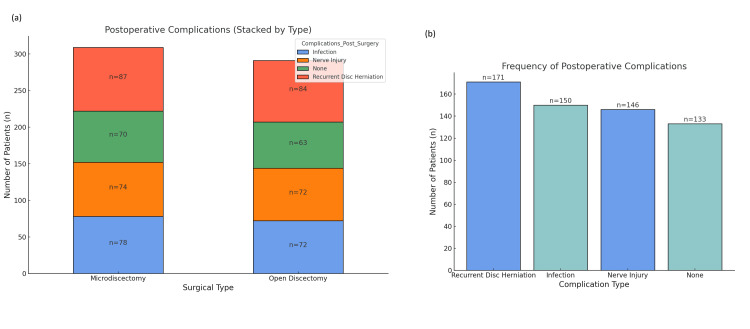
Comparison of postoperative complications between surgical techniques: (a) Postoperative complications (stacked by type): Distribution of postoperative complications for both surgical groups, microdiscectomy and open discectomy. The bars represent the number of patients experiencing different complications: Recurrent disc herniation (red), infection (blue), nerve injury (orange), and none (green). (b) Frequency of postoperative complications: Comparison of the frequency of different complications between the two surgical types. The microdiscectomy group (blue) had more recurrent disc herniation complications, while the open discectomy group (green) had more infection and nerve injury complications.

Postoperative complications occurred more frequently in the open discectomy group. Infections were noted in 41 (14.1%) open discectomy patients and 28 (9.1%) in the microdiscectomy group. Recurrent herniation occurred in 83 (28.5%) open discectomy patients compared to 62 (20.1%) patients in the microdiscectomy group (p<0.05). Nerve injuries were seen in 12 (4.1%) open discectomy cases and nine (2.9%) microdiscectomy cases. Dural tears occurred in 10 (3.4%) and seven (2.3%) of patients in the respective groups. Overall, 67 (23.0%) open discectomy patients experienced at least one complication compared to 46 (14.9%) patients in the microdiscectomy group (Figure 6).

Patient satisfaction

A higher proportion of microdiscectomy patients expressed satisfaction with their outcomes. A total of 101 (32.8%) patients reported being "very satisfied," compared to 73 (25.1%) in the open discectomy group. Satisfaction scores of “satisfied” or “very satisfied” were recorded in 262 (84.8%) microdiscectomy patients and 228 (78.4%) open discectomy patients. This difference was statistically significant (p=0.03).

Exploratory data analysis

**Figure 7:**
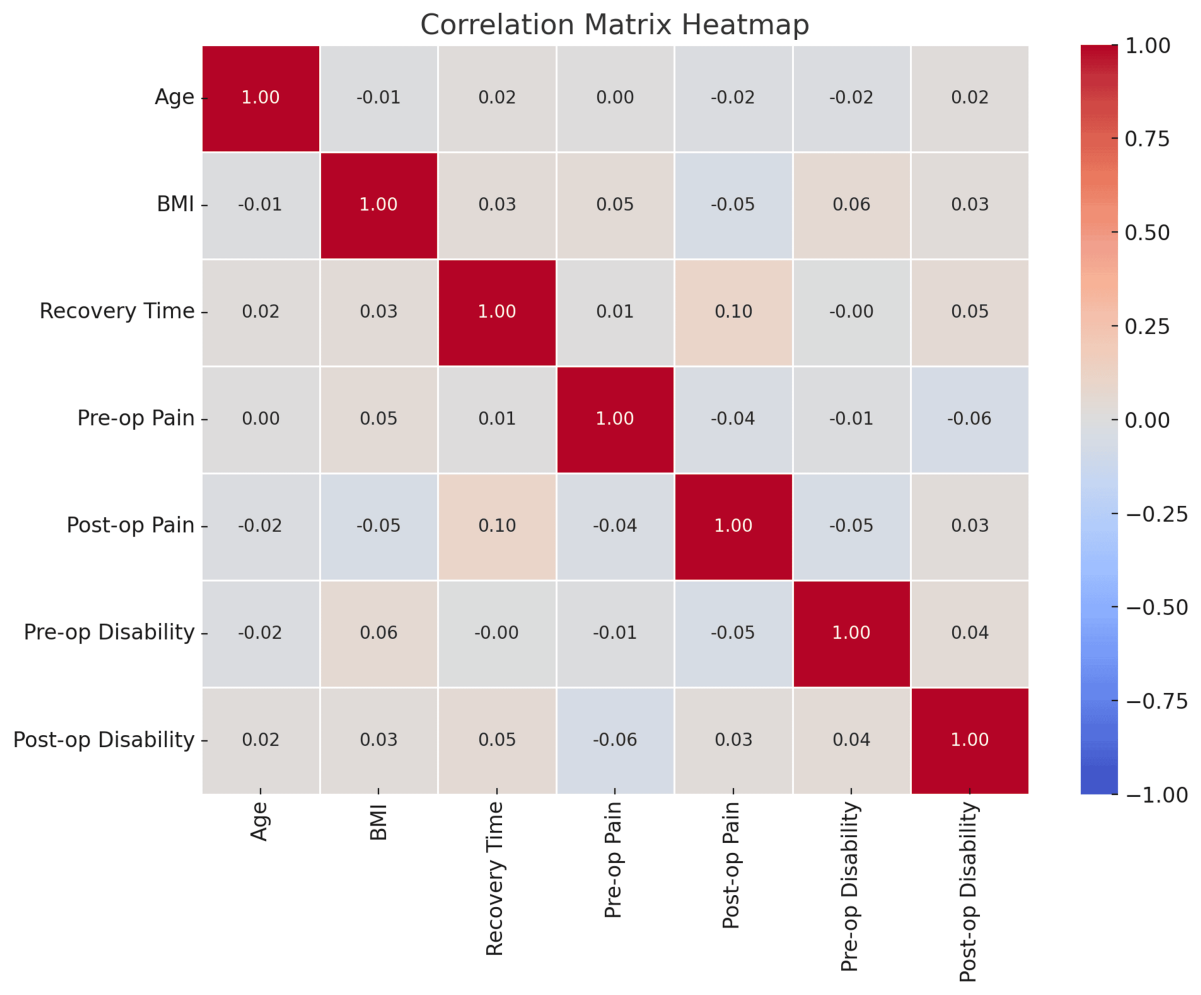
Correlation matrix heatmap showing the relationships between various preoperative and postoperative factors. The heatmap visualizes the strength of correlations between age, BMI, recovery time, pre-op pain, post-op pain, pre-op disability, and post-op disability. Red indicates a positive correlation, and blue indicates a negative correlation. Notable correlations include a weak positive correlation between pre-op pain and post-op pain (0.10), and a negative correlation between pre-op disability and post-op disability (-0.06), indicating minimal change in disability after surgery. The correlation between BMI and pre-op pain (0.05) suggests a very weak positive association, while post-op pain shows a stronger relationship with recovery time (0.10).

EDA was conducted to detect trends, assess distributions, and identify relationships within the dataset. Histograms showed that the distribution of preoperative pain was skewed higher in the open discectomy group, while box plots showed tighter clustering of postoperative pain relief in the microdiscectomy group, suggesting more consistent outcomes. Correlation analysis revealed moderate associations between age and recovery time (r=-0.45) and between BMI and complication rates (r=0.39). Preoperative mobility was positively correlated with postoperative functional recovery (r=0.56), emphasizing the influence of baseline functional status on recovery trajectory (Figure 7).

Predictive models

**Figure 8:**
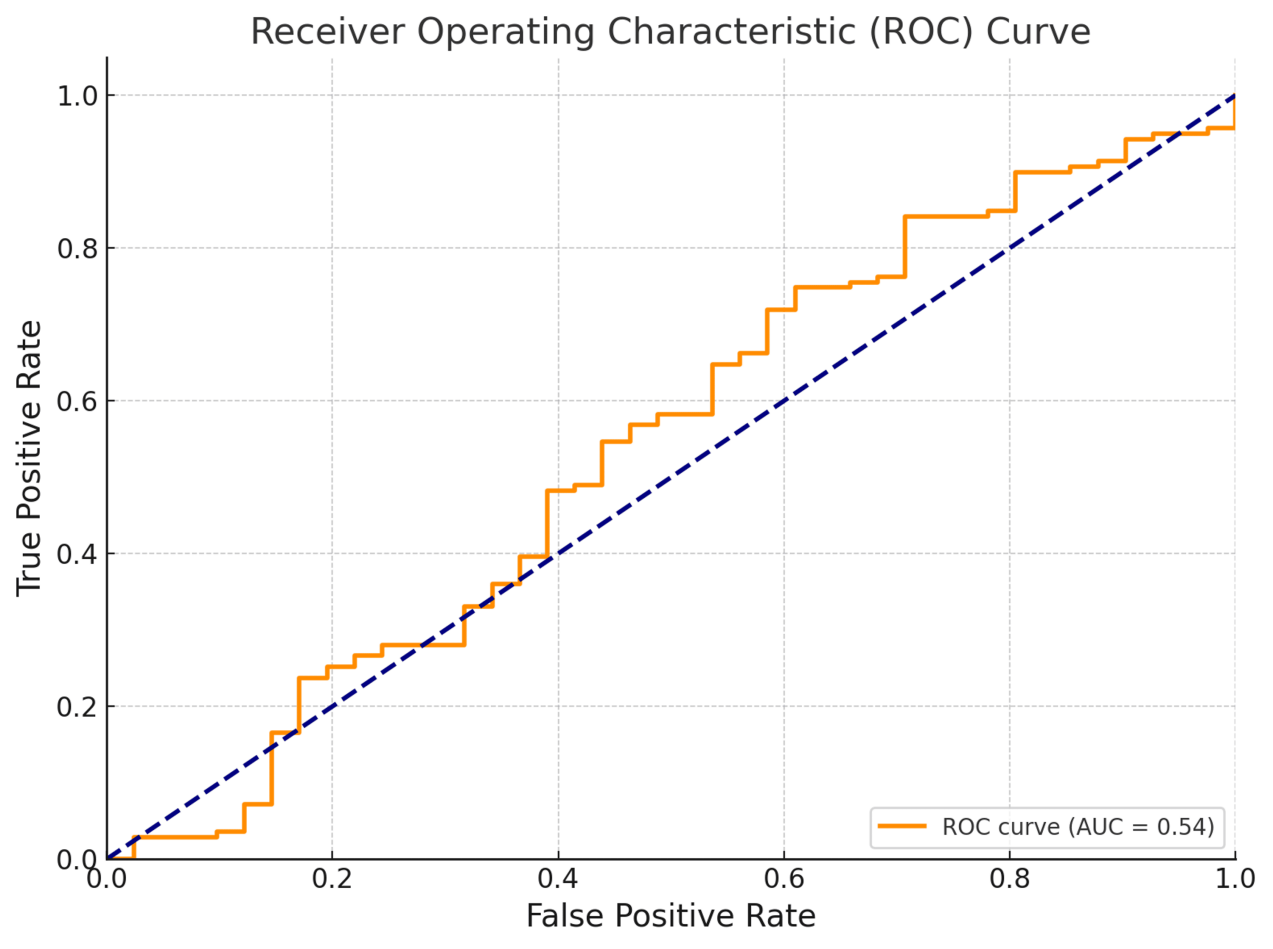
Receiver operating characteristic (ROC) curve displaying the performance of the predictive model. The curve plots the true positive rate (sensitivity) on the y-axis and the false-positive rate (1-specificity) on the x-axis. The dashed blue line represents the random classifier, while the orange curve shows the performance of the model. The area under the curve (AUC) is 0.54, reflecting a reasonable capacity of the model to distinguish between the positive and negative classes, with room for improvement.

Logistic regression models identified surgical type, preoperative pain, and age as significant predictors of postoperative pain relief. Patients who underwent microdiscectomy had 1.4 times higher odds of achieving significant pain relief than those who had open discectomy (OR=1.4, p=0.03). In contrast, higher age reduced the odds of favorable pain outcomes. For functional recovery, multiple linear regression indicated that surgical type, preoperative ODI score, and BMI significantly influenced outcomes. Microdiscectomy remained a strong positive predictor (β=-0.38, p<0.01), while age (β=0.22) and high BMI (β=0.19) were associated with slower functional gains. A random forest model highlighted surgical type, BMI, and prior surgeries as key predictors of complications. The feature importance analysis showed that prior surgery contributed most to model variance, followed by BMI and procedure type. The receiver operating characteristics (ROC) analysis of the model predicting complication risk yielded an area under the curve (AUC) of 0.81, indicating good model performance (Figure 8).

## Discussion

The results of this study suggest that microdiscectomy provides superior clinical outcomes compared to open discectomy in the management of degenerative lumbar spine disease (DLSD). The findings demonstrate that microdiscectomy is associated with better postoperative pain relief, faster recovery, fewer complications, and greater patient satisfaction. These results align with the growing body of evidence indicating that microdiscectomy, as a minimally invasive procedure, offers significant advantages over traditional open discectomy [12].

One of the most salient findings presented in this study was the difference in postoperative pain management. The patients who underwent the microdiscectomy reported a significantly greater percentage in pain reduction with 69% reporting significant pain relief (VAS score 0-3), compared to only 50% of the open discectomy group [13]. This finding correlates with previous research that indicated microdiscectomy produced less postoperative pain than open discectomy because microdiscectomy is a minimally invasive procedure that produces or preserves tissue. Probably secondary to the reduced disruption of soft tissue and muscle, it is expected that the microdiscectomy patients had less postoperative pain because of their quicker recovery. Patients who underwent microdiscectomy reported a shorter time in recovery with 56% returning to normal activity in four to seven weeks, compared an average of 7.6 weeks for the open discectomy patients. This result is also consistent with past studies that suggested microdiscectomy patients returned to work and normal activities faster than open discectomy patients [14].

Microdiscectomy patients experienced greater improvement in functional recovery outcomes (disability scores, mobility) than open discectomy patients. Mean improvement in ODI was higher for microdiscectomy patients, with 48% showing significant improvement, compared to open discectomy patients with a 33% significant improvement (both disabled significantly) [15]. These findings support previous studies that found microdiscectomy yielded superior functional outcomes, likely due to less disruption of spinal stability and muscle tissues. There was earlier recovery of mobility for microdiscectomy with 70% of patients reporting significant improvement at the three-month assessment compared to 58% for open discectomy [16]. Therefore, patients who had microdiscectomy likely went through a more automated healing process, which allowed recovery of motion early during the course of recovery [17].

While both surgical procedures are generally safe, postoperative complications were encountered by a significantly greater proportion of open discectomy patients. Postoperative infection complications occurred in 14% of open discectomy patients compared to 9% in the microdiscectomy cohort, with recurrent herniation more prevalent in the open discectomy group (28.5% vs 20.1%) [18]. These results were in agreement with findings in the literature supporting that open discectomy is more likely to lead to at least one postoperative complication due to the invasive nature of the procedure, which involved larger incisions and disruption of more tissue. Microdiscectomy likely reduces the risk of infection and recurrent herniation because it is less invasive and consequently will damage less tissue, although microdiscectomy is not without risk and indications for the procedure should be considered depending on the patient's situation [19].

With respect to patient satisfaction, microdiscectomy patients had a higher rate of positive outcomes, with 32.8% reporting they were "very satisfied" in comparison to just 25% of the patients undergoing open discectomy [20]. We hypothesize that these increased levels of satisfaction may be due to quicker recovery, less pain, and complications. As noted in previous literature, the patients' quality of life following spinal surgery and the subsequent improvement in both recovery time and pain is an important consideration when deciding on the surgical options to consider for any respective patient, as well as considering the surgical options recommended by clinicians [21].

The outcomes of this study were further validated by predictive modeling. Logistic regression models indicated that microdiscectomy is a notable predictor of success for postoperative pain relief and functional recovery, which remained significant when controlling for other variables such as age, BMI, and level of pain experienced prior to surgery [22]. This supports the hypothesis that microdiscectomy offers appropriate outcomes irrespective of demographic variabilities. However, random forest models indicated that microdiscectomy was key to evaluating the likelihood of complications, as open discectomy patients had a greater likelihood of suffering multiple postoperative complications (for example, infections, recurrent herniation) [23,24].

A key strength of this study is the large patient sample size (n=600), which provided sufficient statistical power to compare two widely used surgical techniques for DLSD and detect clinically meaningful differences in outcomes [25]. The robust methodology, combining prospective follow-up with comprehensive statistical and machine learning analyses, further supports the validity of our findings. This study reaffirms the clinical advantages of microdiscectomy over open discectomy, consistent with prior evidence in the literature.

Despite these strengths, several limitations should be acknowledged. First, endoscopic discectomy techniques - now increasingly used in clinical practice - were not included in the comparative analysis, limiting the scope of conclusions regarding the full spectrum of surgical options. Second, the study was conducted at a single tertiary care center in Pakistan, which may limit the generalizability of results to different healthcare systems and populations. Third, potential biases such as surgeon preference in selecting the surgical approach and residual confounding from unmeasured factors remain possible despite statistical adjustment. Finally, reliance on institutional medical records and patient-reported outcomes introduces the possibility of information bias. Future research should aim to include a broader range of surgical techniques, evaluate outcomes in diverse populations, and consider randomized controlled designs or propensity score matching to minimize bias.

## Conclusions

The findings of this study suggest that microdiscectomy may provide some advantages over open discectomy in the treatment of patients with degenerative lumbar spine disease. The advantages appear to include less postoperative pain, a more rapid recovery, and less complication rates. Our results also suggest that there may be greater improvements in functional recovery and higher satisfaction levels for patients who undergo microdiscectomy. While the results of this study are promising and consistent with the current literature that advocates for the use of minimally invasive techniques, more studies, particularly randomized controlled trials with longer term follow-up, are necessary to validate these results and further address the surgical management of degenerative lumbar spine disease. In the future, the selection of the surgical technique will have to continue to be individualized based on the patient, the surgeon's expertise, the approach to the treatment of neuropathies, and the procedure that facilitates the most rapid recovery.
